# Gut microbiota-associated nutritional-immune status predicts prognosis in postoperative NSCLC patients

**DOI:** 10.1080/19490976.2026.2652460

**Published:** 2026-04-03

**Authors:** Qian Yu, Anqi Chen, Junqi Yi, Majid iqbal, Ziying Tang, Huabo Ge, Yan Hu, Wenliang Liu, Leliang Zheng, Jingqun Tang, Juanjuan Xiang

**Affiliations:** a Hunan Key Laboratory of Early Diagnosis and Precise Treatment of Lung Cancer, The Second Xiangya Hospital, Central South University, Changsha, Hunan, China; b Department of Thoracic Surgery, the Second Xiangya Hospital, Central South University, Changsha, Hunan, China; c NHC Key Laboratory of Carcinogenesis and the Key Laboratory of Carcinogenesis and Cancer Invasion of the Chinese Ministry of Education, Cancer Research Institute, School of Basic Medical Science, Central South University, Changsha, Hunan, China

**Keywords:** NSCLC, prognostic nutritional index, gut microbiota, SCFA, CD8^+^ T cells

## Abstract

**Background:**

Surgical resection is the primary treatment for non-small cell lung cancer (NSCLC) patients with stages I and II; however, the postoperative prognosis varies among individuals. The prognostic nutritional index (PNI) reflects the nutritional-immune status of patients, but its microbial determinants remain unclear.

**Methods:**

PNI was analyzed in a cohort of 372 retrospective and 139 prospective NSCLC patients. This analysis integrated gut microbiota signatures using 16S rRNA sequencing, fecal metabolomics, and murine fecal microbiota transplantation (FMT) models.

**Results:**

A PNI value of ≥46.2 stratified postoperative NSCLC patients with improved 5-y survival (HR = 0.3889, 95% CI 0.2840–0.5356, *p* < 0.001). Patients with a high PNI showed enrichment of short-chain fatty acid (SCFA)-producing taxa, such as *Akkermansia* and *Eubacterium hallii*, and elevated butyrate/isovalerate levels, correlating with increased infiltration of CD8^+^ T cells (Pearson *r* = 0.51, *p* = 0.02). FMT from high-PNI patients reduced lung tumor growth in mice compared with FMT from low-PNI patients (7.2 vs 18 nodules, *p* = 0.01). Oral administration of *A. muciniphila* or/and *E. hallii* or butyrate suppressed tumor growth and enhanced CD8^+^ tumor-infiltrating lymphocytes (TILs) (*p* < 0.001).

**Conclusion:**

PNI and its linked gut microbiota‒SCFA axis are clinically prognostic biomarkers and potential immunomodulatory targets for early-stage NSCLC. Targeting this axis may serve as a promising coadjuvant strategy for NSCLC patients undergoing surgical resection.

## Graphical abstract

**Figure:**
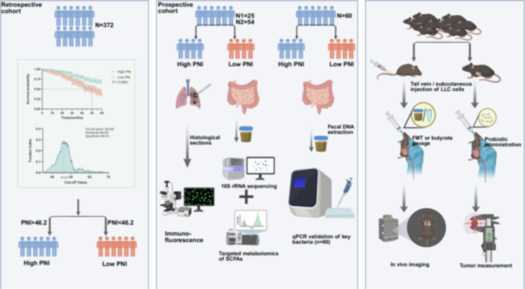

## Introduction

Non-small cell lung cancer (NSCLC), which accounts for 85% of lung cancer, is a leading cause of cancer-related mortality worldwide. While surgical resection and perioperative treatment strategies (e.g., integrated with neoadjuvant) offer curative options for patients with stages I and II, the postoperative outcomes can vary considerably. The TNM staging system serves as a crucial prognostic factor for lung cancer patients after surgery, with other variables such as age, sex, comorbidities, and lung function also impacting patients' outcome.^1-3^ Emerging evidence underscores the value of nutritional and immune status in cancer patients' prognosis, with the prognostic nutritional index (PNI), which is calculated from serum albumin and lymphocyte counts, serving as a practical, cost-effective predictor of survival.^4^^,^^5^ Several studies have linked PNI with the prognosis of cancer patients^6-8^ and shown that PNI is an independent prognostic indicator.^9^ It is a new concept where a clinical characteristic of a patient, not a tumor characteristic, could affect the outcome.^10^ Despite the recognized association between PNI and cancer outcomes, the biological determinants of PNI and its mechanistic links to tumor progression remain incompletely understood. PNI has not been fully integrated into clinical guidelines due to variable cutoff values, susceptibility to acute-phase changes, and lack of interventional validation.^11^ In situations where lymphocyte/albumin levels change rapidly, their effectiveness in guiding interventions requires further testing, and the use of albumin is controversial.^12^

Numerous studies have highlighted the relationship between the immune system and gut microbiota.^13^^,^^14^ The gut microbiome is a complex ecosystem of microorganisms that can influence various physiological processes, including metabolism, immune response, and inflammation. Short-chain fatty acids (SCFAs), produced by the fermentation of dietary fibers by gut bacteria, have been shown to exert anti-inflammatory effects and enhance immune function. Understanding how the gut microbial communities influence nutritional and immune status offers the opportunity to improve the prognosis of lung cancer patients by modulating gut microbiota.

Here, we propose that gut microbiota composition and SCFA production influence NSCLC outcomes through immunometabolic reprogramming. Through the analysis of retrospective and prospective cohorts, we validate PNI as an independent prognostic marker for early-stage NSCLC patients and identified SCFA-producing microbiota signatures predictive of survival. In murine models, targeted probiotic supplementation suggested that *Eubacterium hallii* and *Akkermansia muciniphila* are associated with reduced tumor growth and enhanced CD8^+^ T-cell responses. This study offers initial insights from murine models into how the gut microbiota may influence postoperative outcomes in NSCLC patients, suggesting potential strategies for future therapeutics for NSCLC patients.

## Materials and methods

### Patients characteristics

The study included the retrospective analysis of 372 patients with NSCLC who underwent surgical resection at the Second Xiangya Hospital of Central South University from February 2015 to July 2019. Patients were diagnosed with either adenocarcinoma or squamous cell carcinoma, confirmed through histological examination. Staging were assessed based on the TNM classification of lung cancer according to the 8th edition of the International Association for the Study of Lung Cancer (IASLC) guidelines. Patients were excluded according to the following criteria: (1) Preoperative tumor-related therapy (chemotherapy, radiotherapy, targeted therapy, or immunotherapy); (2) Distant metastasis; (3) History of other tumors or acute or chronic diseases; and (4) Non-lung-cancer-related death. The cohort comprised 190 stage I, 105 stage II, and 77 stage III patients (Supplementary Figure 1). The detailed clinicopathological characteristics are summarized in Supplementary Table 1.

To investigate the association between microbiota-derived metabolites and the PNI, a multiomic prospective analysis of the microbiome and metabolome was conducted on an additional 25 NSCLC patients (cohort 1) and 54 NSCLC patients (cohort 2). Detailed clinicopathological characteristics of this group are summarized in Supplementary Tables 2–3. Fecal samples were collected from these two cohorts without presurgery antibiotic treatment. An additional 60 NSCLC patients were prospectively included to validate the specific bacterial abundance. The detailed clinicopathological characteristics are summarized in Supplementary Table 4.

The prognostic nutritional index (PNI) was calculated by the formula PNI = serum Alb (g/L) + 5 × TLC (×10^9^/L). Overall survival (OS) was defined as the period from the date of surgery to the date of death due to non-small cell lung cancer causes or the date of the last follow-up visit. The ROC curve was constructed based on the predicted probabilities from the logistic regression model, and the optimal cutoff was determined by maximizing the Youden index. Subsequently, cohorts 1 and 2 were both stratified into high-PNI (PNI ≥ 46.2, HPNI) and low-PNI (PNI < 46.2, LPNI) groups, with none of the patients having used antibiotics or systemic therapies before sampling (Supplementary Tables 2 and 3). The analysis included gut microbiota composition and fecal short-chain fatty acid (SCFA) levels. LEfSe analysis was applied to identify differentially abundant taxa between the PNI groups. In addition, an additional 60 newly diagnosed patients were included to evaluate the association of specific species abundance with PNI (Supplementary Table 4).

### Samples collection, 16S rRNA sequencing, and multi-region 16S rRNA gene sequencing (5 R 16S)

Fecal samples from NSCLC patients and experimental mice were collected for subsequent 16S rRNA sequencing analyses. All patient samples were obtained from the Department of Thoracic Surgery, Second Xiangya Hospital of Central South University, and were collected prior to surgical resection, including cohort 1 and an additional cohort 2. Cohort 2 was used for independent validation and additional support of the findings from cohort 1. All the fecal specimens (>5 g) were aseptically collected, immediately frozen at −80 °C, and stored until analysis. Written informed consent was obtained from all participants.

Genomic DNA was extracted according to the manufacturers' instructions: samples from cohort 1 were processed using the Magen Hipure Stool DNA kit (#D314102), whereas samples from cohort 2 and experimental mice were extracted using a TIANGEN kit (Beijing, China, #DP328-02).

For cohort 1, the V3–V4 hypervariable regions of the bacterial 16S rRNA gene were amplified using primers 341F (5′-CCTAYGGGRBGCASCAG-3′) and 805 R (5′-GGACTACNNGGGTATCTAAT-3′), and amplicons were sequenced on an Illumina MiSeq platform (Allwegene Co., China). For cohort 2 and experimental mice, 5 R 16S rRNA gene sequencing was performed on an Illumina NovaSeq 6000 platform (RRID:SCR_016387). High-quality sequences were subjected to reconstruction analysis using the Short Multiregion Framework (SMURF) with the Greengenes database as a reference (RRID:SCR_002830.^15^^,^^16^ The PCR primers used for 5 R sequencing are listed in Supplementary Table 5.

### Gas chromatography–mass spectrometry (GC–MS) analysis

Approximately 20 mg of fecal samples were homogenized in 1 mL of 0.5% phosphoric acid using a steel bead, followed by vortexing and ultrasonication. After centrifugation (12,000 r/min, 10 min, 4 °C), 0.1 mL of the supernatant was mixed with 0.5 mL of MTBE containing an internal standard. The mixture was vortexed, ultrasonicated, and centrifuged again under identical conditions. The resulting supernatant was subjected to GC–MS/MS analysis. An Agilent 7890B gas chromatograph coupled to a 7000D mass spectrometer (Agilent Technologies, USA) equipped with a DB-FFAP capillary column (30 m × 0.25 mm i.d., 0.25 µm film thickness; J&W Scientific, USA) was used. Helium served as the carrier gas at a flow rate of 1.2 mL/min, with a 2 µL split injection. The oven temperature was programmed from 90 °C through sequential ramps to 200 °C, with final holding times as described. The injector and transfer line temperatures were set at 200 °C and 230 °C, respectively. Data were acquired in multiple reaction monitoring mode.

### Liquid chromatography–mass spectrometry(LC–MS)-based metabolomics analysis

Fecal samples collected from the animal experiments were stored at −80 °C and thawed on ice. A 400 μL solution (methanol:water = 7:3, V/V) containing an internal standard was added to a 20 mg sample, and then vortexed for 3 min. The sample was sonicated in an ice bath for 10 mins and vortexed for 1 min, and then placed in −20 °C for 30 min. The sample was then centrifuged at 12,000 rpm for 10 min (4 °C), and the sediment was removed. A 200 μL aliquots of the supernatant were collected finally and examined using LC–MS analysis according to the standard procedures.

### 16S rDNA quantitative PCR (qPCR)

Stool DNA was extracted with the HiPure Stool DNA Kit (Magen, D314102) and quantified on a NanoDrop spectrophotometer (RRID:SCR_018042). Species-specific primers were designed with NCBI Primer-BLAST against discriminatory regions of the 16S rRNA gene (V1/V2), while a universal 16S primer pair targeting a conserved region served as the total-bacteria reference; in-silico coverage and specificity were verified with the SILVA TestPrime, and amplicon identity was supported by single-peak melt curves. Reactions (20 µL; SYBR Green Pro Taq HS, Accurate Biology AG11701; primers 400 nM) were run with initial denaturation at 95 °C for 30 s, followed by 40 cycles of 95 °C for 5 s and 60 °C for 30 s, then with melting curve analysis. Relative abundance calculation was computed as follows: (*i*) ​= *2^−ΔCT(ΔCt = Ct(target) − Ct(universal 16S))^.*^17^ Final primer sequences are provided in Supplementary Table 6.

### Lewis lung carcinoma-luciferase (LLC-Luc) mouse model

Six-week-old C57BL/6 mice were purchased from Slake Experimental Animal Co., Ltd. (China, Shanghai). When injected with luci-LLC (CVCL_E3HS), mice were detected by in vivo imaging 3 weeks later. All animal experiments were conducted following protocols approved by Central South University, China. We injected 1 × 10^5^ LLC-luc cells into 8- to 10-week-old mice, and the clinical endpoint was achieved when the mice exhibited signs of expiratory dyspnea.

### Fecal microbiota transplantation (FMT)

Fresh stools from NSCLC donors with high-PNI or low-PNI patients were processed within ≤2 h under anaerobic conditions. Approximately 2.5 g of stool was suspended in 10 mL of prereduced PBS, homogenized, passed through a 70-µm strainer, clarified at 800 × *g* for 3 min, pelleted at 3000 × g for 10 min, and resuspended in 2 mL of PBS; 200 µL aliquots were used immediately for gavage. C57BL/6J mice (6 weeks) were treated with an antibiotic cocktail in the drinking water for 14 d: ampicillin (0.2 g/L), vancomycin (0.1 g/L), neomycin (0.2 g/L), and metronidazole (0.2 g/L). Mice were randomized to HPNI_FMT, LPNI_FMT, or PBS (No_FMT) and gavaged with 200 µL twice weekly from week −2 to week 0 (four doses). Fresh fecal pellets were collected at week −2 (pre-FMT) and week 0 (post-FMT) for 16S-based engraftment assessment. At week 0, all the mice were induced to develop lung cancer via tail-vein injection of LLC-luciferase cells (1 × 10^5^ cells in 100 µL PBS). At week 6, all the mice were sacrificed, and tumor tissue was collected for further test.^17^

### Gavage experiments with sodium butyrate

Mice received sodium butyrate (Sigma, #303410) dissolved in sterile PBS at 2 mg/mL by oral gavage at 200 μL per mouse once daily; the vehicle controls received equal volumes of PBS. Gavage started 2 weeks before tumor inoculation and continued thereafter. At week 0, all the mice were injected via the tail vein with LLC-luciferase cells (1 × 10^5^ cells in 100 μL of PBS). At week 6, the mice were euthanized, and tumor tissues were collected for downstream analyses.^17^

### Gavage experiments with SCFA-producing bacteria

The mice were randomly divided into four groups (PBS/Amu/Eha/Amu + Eha) (Amu: *Akkermensia muciniphila* (ATCCBAA-835), Eha: *Eubacterium hallii* (DSM3353)). We carried out mouse studies with SCFA-producing bacteria (1*10⁹ CFU/mL) by gavage, with the control being with PBS. Each group of mice was given 200  μL by gavage once a day. After 21 d of gavage, the mice were induced to develop lung cancer by subcutaneous injection of LLC (CVCL_4358) cells (1 × 10^5^ cells in 100 μL PBS), and afterwards all the mice still received gavage daily. When subcutaneous tumors formed, it was marked as ay 1. The mice were sacrificed on day 11, and tissue was collected for further test.

### H&E staining

Tissue sections were stained with Harris' hematoxylin for 3–5 min at room temperature. Subsequently, they were rinsed in running tap water for 3–5 min. To differentiate the tissue, 1% acid alcohol (1% HCl in 70% ethanol) was applied for 3–10 s under microscopic control, followed by a tap-water rinse. Sections were then blued in saturated lithium carbonate for 30–60 s and rinsed with tap water. Finally, they were counterstained with eosin Y (0.5%–1.0%) for 30–60 s, dehydrated through graded ethanol, cleared in xylene, and mounted.

### Immunofluorescence staining

Tumor samples from animal were collected. The 4 μm-thick slides cut from the FFPE blocks were dewaxed with an eco-friendly dewaxing solution. Then, the tumor sections were deparaffinized, rehydrated, and stained using the following antibodies: CD8 (RRID: AB_2756376) (Cell Signaling Technology, 989411). Ultimately, the slides were stained for ten minutes using 4′-6′-diamidino-2-phenylindole (DAPI).

### Statistical analysis

Statistical analyses were performed using SPSS (RRID:SCR_002865) and GraphPad Prism (RRID:SCR_002798) software. Continuous variables were compared using the independent samples *t* test or ANOVA, while categorical variables were analyzed with the chi-square test or rank sum test. The primary endpoints, OS were assessed by Kaplan‒Meier curves. Prognostic factors were evaluated through univariable and multivariable Cox proportional hazard regression models, with the results presented as hazard ratios (HRs) and 95% confidence intervals. The prognostic performance of the PNI for 5-y survival was evaluated using receiver operating characteristic (ROC) curve, with the optimal cutoff value determined by Youden's index. A two-sided *p* value less than 0.05 was considered statistically significant.

Statistical analyses for differential metabolites were performed using the R package MetaboAnalystR (RRID:SCR_016723). Alpha diversity indices (Shannon, Simpson, Chao1) and beta diversity metrics (Bray–Curtis, weighted UniFrac) were calculated. Community differences were assessed by principal component analysis (PCA/PCoA) and analysis of similarities (ANOSIM; RRID:SCR_002823). Differentially abundant taxa were identified using LEfSe (RRID:SCR_016098) with an LDA score > 2.0 and FDR-adjusted *p* < 0.05. Data were log-transformed (log2) and mean-centered prior to performing orthogonal partial least squares discriminant analysis (OPLS-DA). The robustness of the OPLS-DA model was validated through a permutation test with 200 permutations. Identified metabolites were annotated using the KEGG Compound database (RRID:SCR_012773) (http://www.kegg.jp/kegg/compound/), and pathway enrichment analysis was subsequently conducted using the KEGG Pathway database (RRID:SCR_018145) (http://www.kegg.jp/kegg/pathway.html).

## Results

### PNI is an independent prognostic factor for early-stage postoperative NSCLC patients

**Figure 1.**
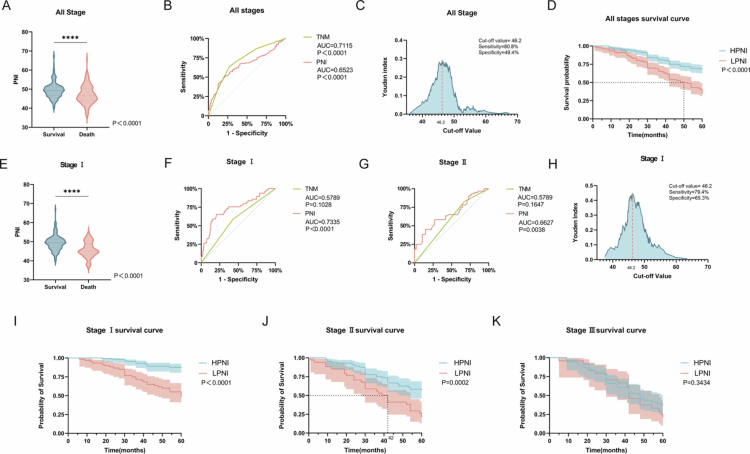
PNI is a independent prognostic factor for early-stage postoperative NSCLC patients. (A) The PNI levels in patients who survived for 5 y and those in deceased patients. (B) The logistic regression model of ROC curves across all clinical stages. (C) Youden index with maximal sensitivity and specificity. (D) Cumulative survival rates of patients across all clinical stages. (E) The PNI levels in clinical stage I patients who survived for 5 y and those in deceased patients. The logistic regression model of ROC curves in clinical stages I (F) and II (G). (H) Youden index with maximum sensitivity and specificity in patients with stage I. Cumulative survival rates of patients with stage I (I), stage II (J) and stage III (K).

In a retrospective cohort of 372 postoperative NSCLC patients, the mean PNI was 48.47 ± 4.71, ranging from 35.40 to 67.70. Patients with 5-year survival exhibited significantly higher PNI levels than nonsurvivors (49.5 vs. 47.1, *p* < 0.0001, Figure 1A). Multivariable cox regression confirmed PNI (HR = 0.3889, 95% CI 0.2840–0.5356, *p* < 0.0001), gender (HR = 1.7310, 95% CI 1.152–2.664, *p* = 0.0101) and TNM stage (HR = 0.3693, 95% CI 0.2515−0.5348, *p* < 0.0001) as independent prognostic factors (Table 1). However, tumor size was shown as an independent prognostic factor in univariable cox regression, but not in multivariable cox regression, suggesting the influence of TNM on the significance of tumor size (HR = 0.7117, 95% CI 0.4638−1.069, *p* = 0.1097). The ROC curve was constructed based on the predicted probabilities from the logistic regression model, with the adjusted ROC curve for PNI demonstrating an AUC of 0.6523 (*p* < 0.0001, Figure 1B), compared to TNM AUC of 0.7115 (*p* < 0.0001, Figure 1B). The optimal PNI cutoff of 46.2, determined by maximizing Youden's index, yielded the highest sensitivity (80.8%) and specificity (49.4%) (Figure 1C). Patients stratified by PNI ≥ 46.2 had superior 5-year survival. The median overall survival (mOS) was not reached in the high-PNI group, whereas it was 50 months in the low-PNI group (95% CI, 42–59; *p* < 0.0001; Figure 1D). We further examined variations in the PNI cutoff value among patients with clinical stage I. Among stage I patients (5-year survival rate, 74.2%), survivors had a significantly higher PNI than nonsurvivors(*p* < 0.0001, Figure 1I, Figure 1E). PNI outperformed TNM staging in prognostic efficacy for stage I (AUC = 0.7335 vs. 0.5789, *p* < 0.0001, Figure 1F) and stage II (AUC = 0.6627 vs. 0.5789, *p* = 0.0038, Figure 1G). Multivariable cox regression for stage I also confirmed that PNI (HR = 0.1986, 95% CI: 0.1037−0.3657, *p* < 0.0001) performed better than TNM (HR = 0.6338, 95% CI: 0.3509−1.129, *p* = 0.1237) (Supplementary Table 7). A PNI cutoff value of 46.2 appears effective across all clinical stages, particularly for stage I patients, where it demonstrated a higher specificity (65.3%) compared to those at other stages (Figure 1H). Survival analysis confirmed prolonged OS in high-PNI stage I (*p* < 0.0001) and stage II (*p* = 0.0002) patients, but not in stage III (*p* = 0.3434) (Figure 1I–K). These findings underline the robust prognostic potential of PNI as a stratification tool for I−II stage postoperative NSCLC prognosis.

**Table 1.** Independent prognostic factors for postoperative NSCLC patients.

	Univariable Cox regression			Multivariable cox regression		
Variable	HR	95% CI	*p*	HR	95% CI	*p*
**Gender**						
Male/female	1.801	1.243−2.685	0.0027*	1.731	1.152−2.664	0.0101*
**Age(years)**						
	1.001	0.9848−1.018	0.9095	1.002	0.9853−1.020	0.8315
**PNI**						
≥46.2/ < 46.2	0.381	0.2786−0.5213	<0.0001*	0.3889	0.2840−0.5356	<0.0001*
**pTNM classification**						
Ⅰ/>Ⅰ	0.3326	0.2353−0.4634	<0.0001*	0.3693	0.2515−0.5348	<0.0001*
**Primary tumor size**						
T1/>T1	0.4697	0.3179−0.6759	<0.0001*	0.7117	0.4638−1.069	0.1097
**Pathological type**						
Adenocarcinoma/squamous cell carcinoma and adenosquamous carcinoma	0.744	0.5441−1.017	0.0633	0.7961	0.5627−1.130	0.199
**Tumor location**						
Left lung/right lung	1.063	0.7773−1.453	0.6998	1.078	0.7844−1.479	0.6432
**Lobe number**						
Single/multiple	0.395	0.2191−0.8029	0.0046*	0.5681	0.3097−1.168	0.0916

^*^ indicates statistical significance (*P < 0.05*).

### Patients with different PNI have distinct gut microbiome composition

**Figure 2.**
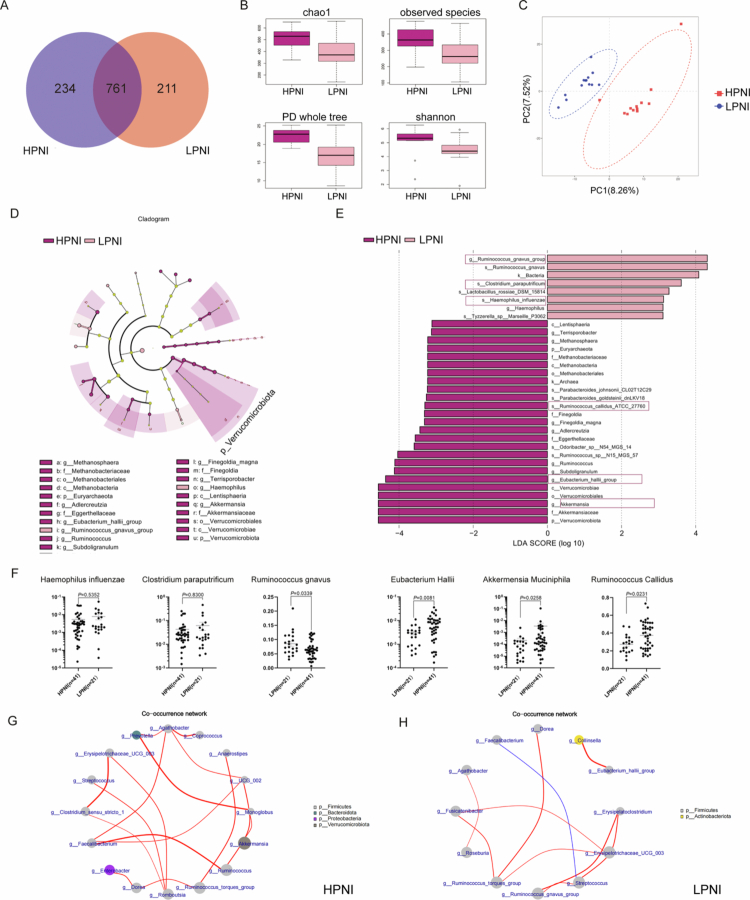
Gut microbiota profiles in NSCLC patients stratified by PNI. The 16S rRNA sequencing was performed to examine the differential bacteria in the high-PNI and low-PNI groups. (A) Venn diagram of shared and unique OTUs in the gut microbiota of patients with between the high-PNI and low-PNI group; (B) Alpha-diversity indices (Chao1, observed species; PD whole tree; Shannon index). (C) PLS-DA plot showing compositional divergence. (D) Cladogram of differentially abundant taxa (LEfSe). (E) LDA scores for discriminating genera (threshold > 2). (F) qPCR validation of key bacterial species. (G and H) Genus-level interaction networks in the high-PNI (G) and low-PNI (H) groups.

Given the pivotal role of the gut microbiota in modulating host nutritional and immune status, we investigated the relationship between the PNI and the gut microbiota composition. We analyzed the gut microbiome profiles in two prospective cohorts (cohort 1: *n* = 25, high-PNI: 12 vs. low-PNI:13; cohort 2: *n* = 54, high-PNI: 37 vs. low-PNI: 17; Supplementary Tables 2−3). We performed 16s rRNA sequencing to examine the gut microbiota composition in cohort 1. We identified 1206 operational taxonomic units (OTUs), with 761 OTUs shared between the high-PNI and low-PNI groups (Figure 2A). High-PNI patients exhibited significantly greater alpha diversity in various metrics, including Chao1, observed species, PD_whole_tree, and Shannon index. The results indicated a significantly higher diversity in patients with high-PNI (Figure 2B). Partial least squares discriminant analysis (PLS-DA) revealed distinct compositional separation between the high-PNI and low-PNI groups, confirming PNI-driven microbiome compositional differences (Figure 2C). Bar plots at both the phylum and genus levels revealed differences in bacterial abundance (Supplementary Figure 2). At the phylum level, high-PNI patients exhibited increased *Verrucomicrobiota*, while low-PNI patients showed enrichment of *Fusobacteriota* (Supplementary Figure 2A). Genus-level analysis highlighted elevated SCFA-producing genera *Akkermansia*, *Eubacterium hallii* group, and *Ruminococcus* in the high-PNI cohort (Supplementary Figure 2B). LEfSe analysis identified specific genera (LDA score > 2) as potential biomarkers for high PNI, including *Akkermansia*, *Eubacterium_hallii*_group, *Ruminococcus*, and others (Figure 2D). The plot of LDA scores highlighted microbial taxa exhibiting significant differences between the two groups (Figure 2E). The LDA scores indicated that the genera *Akkermansia*, *Eubacterium_hallii_group*, *Ruminococcus*, *Subdoligranulum*, *Sellimonas*, *Adlercreutzia*, *Finegoldia_magna,* and *Methanosphaera* were enriched in the high-PNI group, while *Haemophilus influenzae*, *R. gnavus,* and *Clostridium paraputrificum* were enriched in the low-PNI group (Figure 2E). These specific taxa, identified by LEfSe with LDA scores greater than 2, can be considered potential biomarkers to distinguish the gut microbiome composition between high-PNI and low-PNI patients. Validation of these findings through bacterium-specific qPCR confirmed higher abundance of certain bacteria in high-PNI patients, such as *E. hallii*, *Akkermensia muciniphila* and *R. callidus*, while lower PNI patients had higher levels of *Haemophilus influenzae*, *Clostridium paraputrificum* and *R. gnavus* (Figure 2F). Cooccurrence network analysis revealed divergent microbial interactions. High-PNI microbiota showed tight phylum-level associations between *Verrucomicrobiota* and *Bacteroidota* (Figure 2G), with dense genus-level connections between *Ruminococcus, Faecalibacterium*, and *Bifidobacterium* (Figure 2G). Unique taxon pairs included *Streptococcus-Romboutsia* (high-PNI) and *Streptococcus-Ruminococcus gnavus* (low-PNI) (Figure 2H). These results establish PNI-associated gut microbiome signatures linked to host immunometabolic status.

In order to identify the species-level differential gut microbiota composition, we performed **5R** 16s rRNA gene **sequencing** in cohort 2 that seqencing 5 specific hypervariable regions of the 16s gene simultaneously. We confirmed the PNI-associated microbiome signature: the high-PNI group showed higher species richness and exhibited an overall separation in community composition from the low-PNI group; moreover, cohort 2 provided species-level support for selected key taxa, particularly *A. muciniphila*, *R. callidus*, and *Clostridium paraputrificum* (Supplementary Figure 3).

### Identification of enterotypes in high-PNI and low-PNI patients

In order to explore the relationship between enterotypes and the immune-nutritional status of NSCLC patients, we conducted phylogenetic profiling using 16S rRNA sequencing data mapped to reference genomes. Principal component analysis (PCA) stratified NSCLC patients into 2 distinct clusters, each corresponding to a distinct enterotype: *Bacteroides*-dominated (Enterotype 1) and *Ruminococcus*-dominated (Enterotype 3) (Supplementary Figure 4A). The Calinski–Harabasz (CH) index was used to determine the optimal cluster number, which verified two different enterotypes (Supplementary Figure 4B). The main contributors to each enterotype were recognized and are shown in Supplementary Figure 4C. *Ruminococcus* mainly contributes to cluster 1, and Bacteroides chiefly contributes to cluster 2. In the enterotype dominated by *Ruminococcus*, high-PNI patients made up 61.54%, while low-PNI patients comprised 38.46%. Conversely, in the *entero* type dominated by Bacteroides, the majority of patients had low PNI (Supplementary Figure 4D). Notably, we observed variation in the abundance of the Rominococcus genus between species (Figure 2E, F). Our analysis revealed that *R. gnavus* was reduced in the high-PNI group, while other *Ruminococcus* species were increased in the same group (Figure 2E, F). This observation implies a potentially adverse role of *R. gnavus* in the nutritional and immune status of NSCLC patients.

### Characterizing the gut microbiome-related SCFAs

**Figure 3.**
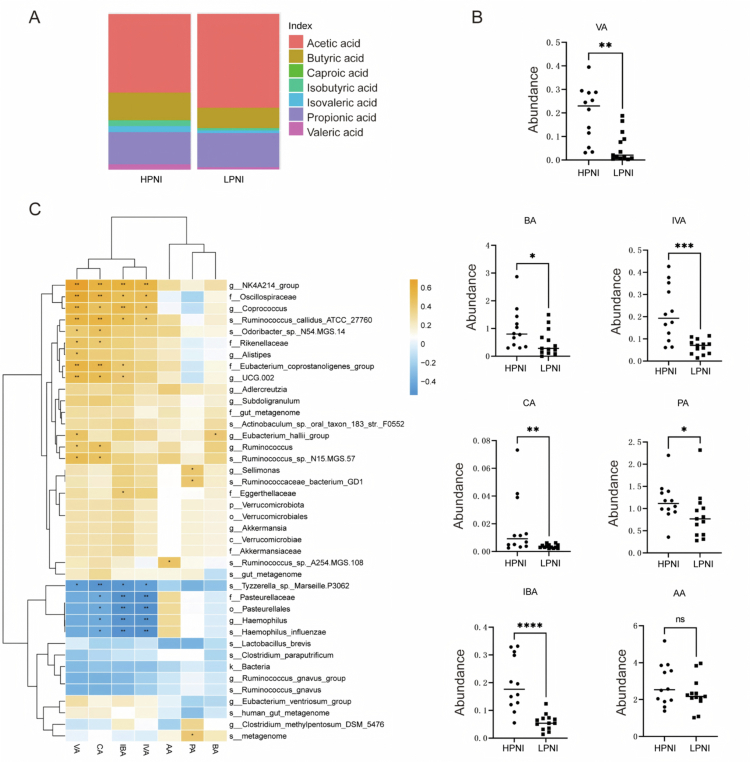
Gut microbiome-linked SCFA production in NSCLC patients. The targeted metabolomics was performed to investigate the absolute values of SCFAs in fecal samples of the high-PNI and low-PNI patients. (A and B) Fecal SCFA concentrations stratified by PNI: (C) Correlation heatmap between microbial genera and SCFAs (Pearson's *r*; red: positive, blue: negative). *p < 0.05, **p < 0.01, ***p < 0.001, and ****p < 0.0001.

To assess the functional link between PNI-associated microbiota and host metabolism, we performed targeted quantitative metabolomics on fecal samples from cohort 1 stratified by PNI. Given the enrichment of SCFA-producing taxa, including *E. hallii, A. muciniphila,* and *R. callidus*, in high-PNI patients, we hypothesized that SCFA levels would be elevated. Accordingly, we observed significant higher levels in isobutyric acid (IBA, *p* = 0.0004), isovaleric acid (IVA, *p* = 0.001), valeric acid (VA, *p* = 0.002), butyric acid (BA, *p* = 0.03), propionic acid (PA, *p* = 0.039), and caproic acid (CA, *p* = 0.004) in high-PNI patients compared to those in low-PNI patients (Figure 3A, B). Pearson's correlation analysis revealed a positive correlation between the genus *Eubacterium_halii* group and both BA (*p* = 0.043) and VA (*p* = 0.025, Figure 3C), while *Ruminococcus* exhibited broad correlation with VA (*p* = 0.038), CA (*p* = 0.023), IBA, IVA, and AA (Figure 3C). Conversely, the abundance of *Haemophilus influenzae,* which was enriched in low-PNI patients, exhibited a significant negative correlation with CA (*p* = 0.04), IBA (*p* = 0.008), and IVA (*p* = 0.005). These findings shed light on the role of the gut microbiota by the production of SCFAs, providing valuable insights into the prognostic factor for NSCLC patients.

### SCFA-producing gut bacteria associate with CD8^+^ T cell infiltration in NSCLC

**Figure 4.**
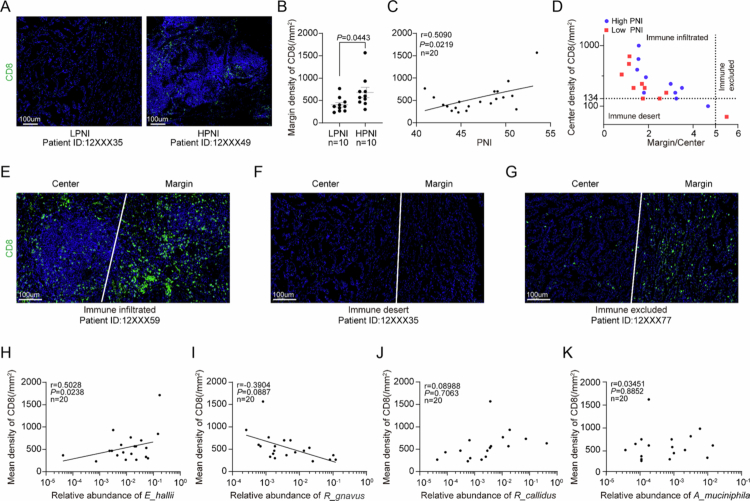
Gut microbiota-CD8^+^ T cell axis in NSCLC. (A) Representative immunofluorescence images of CD8^+^ T cells (green) in tumor sections. Nuclei were counterstained with DAPI (blue). Scale bar: 50 μm. (B) Quantification of CD8^+^ T cell density stratified by the PNI. (C) Correlation between the PNI and CD8^+^ TIL density. (D) Immune phenotypes based on CD8⁺ distribution in the tumor center vs. margin. (E–G) Representative immune-infiltrated, immune-desert, and immune-excluded phenotypes. (H–K) Correlation of CD8^+^ T cell density with gut microbes: *Eubacterium hallii* (H), *Ruminococcus gnavus* (I), *Ruminococcus callidus* (J), and *Akkermansia muciniphila* (K).

As tumor-infiltrating lymphocytes (TILs) play a crucial role in controlling the growth and spread of cancer cells, we investigated the relationship between the PNI, the gut microbiota and CD8^+^ TILs. Immunofluorescence analysis revealed that the density of CD8^+^ T cells in tumor tissues was significantly higher in high-PNI patients compared to the low-PNI cohort (*p* = 0.04, Figure 4A, B). Additionally, there was a positive correlation between PNI scores and CD8^+^ TIL density (Pearson's *r* = 0.51, *p* = 0.02, Figure 4C). Additionally, we observed a positive association between TILs and PNI levels (Figure 4C). Spatial profiling classified 90% (18/20) of the tumors as ‘immune-infiltrated’ (CD8^+^ T cell density ≥25th percentile in the tumor core) (Figure 4D–G). Strikingly, the abundance of SCFA-producing *E. hallii* correlated with CD8^+^ T cell density (*r* = 0.50, *p* = 0.02), while *R. gnavus* exhibited an inverse trend (*r* = −0.39, *p* = 0.089, Figure 4H–K), suggesting that gut microbiota-derived SCFAs may shape an immunopermissive tumor microenvironment conducive to CD8^+^ T cell-mediated antitumor responses.

### SCFA-producing bacteria and metabolite suppress tumor progression

**Figure 5.**
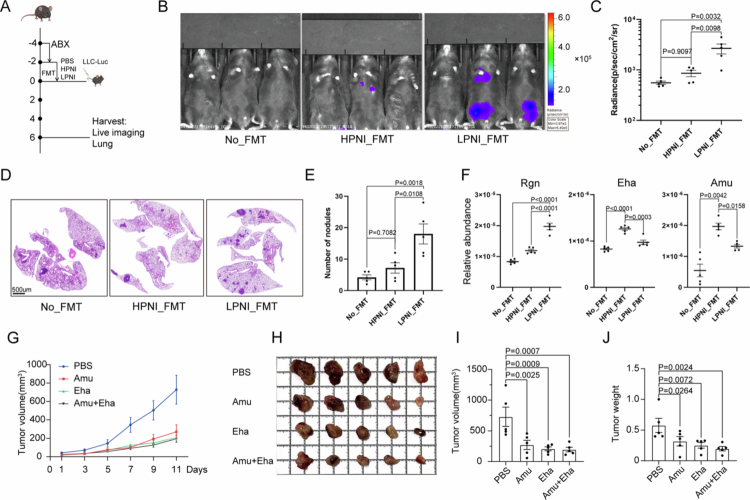
Antitumor effects of SCFA-producing bacteria and metabolites. (A) FMT experimental workflow. (B) Bioluminescence images of LLC metastases. (C) Quantification of tumor flux (photon/sec/cm²). (D and E) H&E-stained lung sections (D) and tumor nodule counts (E). (F) qPCR quantification of donor-derived bacteria in recipient mice. (G) Subcutaneous LLC tumor growth curves. (H) Representative images of excised tumors at the endpoint (day 11). (I) Quantification of tumor volume at the endpoint. (J) Tumor weight quantification. Data: mean ± SEM; *p < 0.05, **p < 0.01, ***p < 0.001, and ****p < 0.0001, (two-way ANOVA was used for (G)); one-way ANOVA with Tukey's post hoc test was used for (C), (E), (F), (I), and (J).

To examine the differential effects of the gut microbiota from high-PNI and low-PNI patients with cancer, we performed fecal microbiota transplantation (FMT) from high-PNI and low-PNI NSCLC patients into antibiotic-pretreated C57BL/6 mice (Figure 5A). The patients were chosen according to their fecal microbiota composition (Supplementary Table 8). Prior to the transplantation of the fecal microbiota from high-PNI and low-PNI patients, the mice were treated with broad-spectrum antibiotics (ABX) to deplete their existing microbiota. Following fecal microbiota transplantation, syngeneic lung cancer cell luciferase-labeled LLCs were intravenously injected. Compared to the FMT from low-PNI patient, FMT from high-PNI NSCLC patients into antibiotic-pretreated mice significantly suppressed lung tumor progression, as evidenced by reduced bioluminescence intensity (*p* = 0.0098) and fewer metastatic lung nodules (7.2 ± 3.701 vs. 18 ± 7.106 in low-PNI FMT, *p* = 0.0108) at week 6 postinoculation (Figure 5B–E). Histological examination, as evidenced by HE staining, displayed fewer tumor nodules in the lungs of mice transplanted with fecal samples from high-PNI patients (Figure 5D, E). However, FMT with the high-PNI patient did not demonstrate tumor shrinkage Compared to mice without transplantation. We then performed 5R 16sRNA sequencing to examine the gut microbiota composition after FMT transplantation. Compared with the group without FMT, the FMT group showed lower alpha diversity and a less connected co-occurrence network, suggesting a potential disruption in microbial community stability following transplantation from lung cancer patients (Supplementary Figure 5A, H). The overall microbial community structure and taxonomic composition differed among the HPNI_FMT, LPNI_FMT, and No_FMT groups, as illustrated by OTU overlap, PCoA ordination, stacked bar plots, and LEfSe analyses (Supplementary Figure 5B–G). Notably, *A. muciniphila* was significantly enriched in HPNI_FMT recipients, which was consistent with the donor-associated signature (Supplementary Figure 5G). qPCR validated successful engraftment of donor-specific microbes, showing elevated *A. muciniphila* (1.48-fold, *p* = 0.0003) and *E. hallii* (1.38-fold, *p* = 0.0158) in HPNI_FMT mice, while LPNI_FMT enriched *R. gnavus* (1.66-fold, *p* < 0.0001) (Figure 5F). We then investigated the roles of specific gut microbes *Eubacterium_halii* and *A. muciniphila* in cancer progression. Oral administration of *Akkermensia muciniphila* or *E. hallii* or their combination starting 14 d preinoculation, followed by subcutaneous injection of LLC cells in C57BL/6 mice (Supplementary Figure 6A). The tumor size was measured every 2 d, and the tumor volume was shown in Figure 5G. The tumor growth was decreased in *A. muciniphila* or *E. hallii. Akkermensia muciniphila* monotherapy reduced the tumor volume by 62% (*p* = 0.0025), and *E. hallii* by 71% (*p* = 0.0009) at day 11, with combinatorial treatment achieving 73% suppression (*p* = 0.0007) (Figure 5G–J).

To examine the role of butyrate in tumor progression, we orally administered a metastatic LLC mouse model that was constructed by tail-vein injection of luciferase-labeled LLC (Supplementary Figure 6B). In a metastatic LLC model, oral butyrate supplementation mimicked HPNI_FMT effects, reducing the lung tumor burden and extending the median survival from 34 to 57 d (*p* = 0.0348) (Supplementary Figure 6C–G). These data demonstrate that SCFA-producing gut bacteria and their metabolites collectively inhibit NSCLC progression through microbiota‒host crosstalk.

### SCFA-producing bacteria modulate gut microbiota composition and enhance TIL infiltration

**Figure 6.**
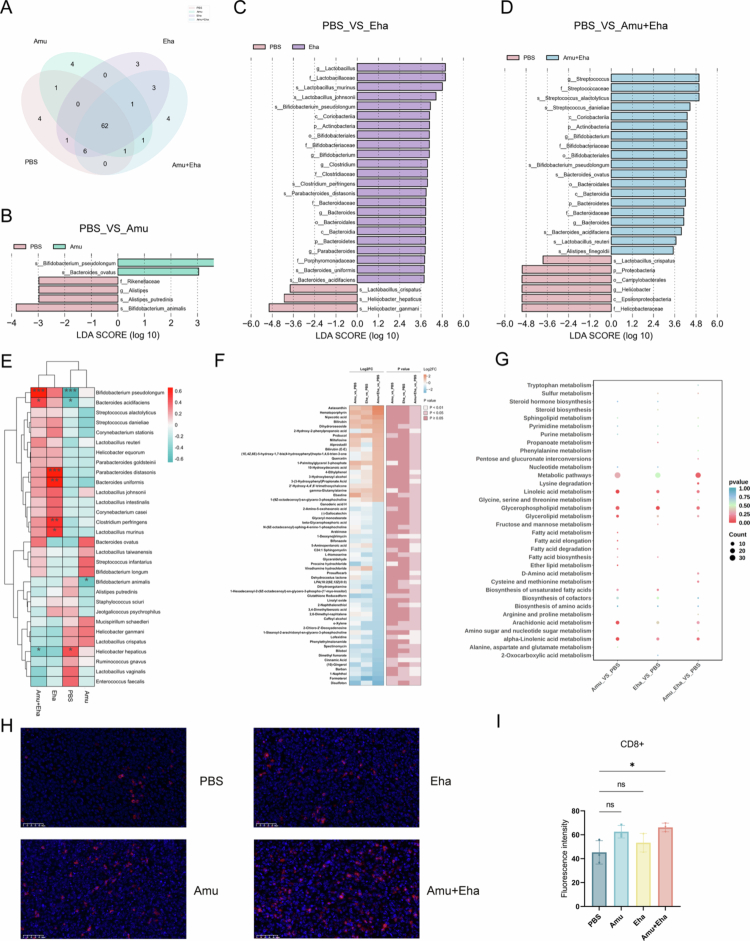
Microbiome, metabolomic, and CD8^+^ T-cell changes after the administration of SCFA-producing bacteria. (A) Venn diagram of shared and group-specific OTUs among groups. (B–D) LEfSe LDA score plots showing differentially enriched taxa in the PBS vs Amu (B), PBS vs Eha (C), and PBS vs Amu + Eha (D). (E) Heatmap and hierarchical clustering of differential species abundances among the PBS, Amu, Eha, and Amu + Eha groups. (F) Heatmap of differential metabolites profiled by LC–MS (pairwise comparisons as indicated). (G) KEGG pathway enrichment bubble plot of differential metabolites (Amu vs PBS, Eha vs PBS, and Amu + Eha vs PBS). (H) Representative immunofluorescence images of intratumoral CD8^+^ cells (red, CD8; blue, DAPI) in the PBS, Amu, Eha, and Amu + Eha groups. (I) Quantification of CD8^+^ fluorescence intensity. Data are presented as mean ± SEM; *p < 0.05, **p < 0.01, ***p < 0.001, and ****p < 0.0001. Statistical analysis: one-way ANOVA with Tukey's post hoc test was used for (I); LEfSe was used for (B–D).

To investigate the impact of SCFA-producing bacteria on the gut microbiota and metabolites, we conducted 5 R rRNA sequencing and untargeted metabolomics on fecal samples from mice treated with *Akkermensia muciniphila*, *E. hallii,* their combination, or negative control. Analysis of 5R sequencing data revealed that 62 gut microbes were shared between the treated and control groups (Figure 6A). Permutational multivariate analysis of variance (PERMANOVA) showed no significant differences in microbial community composition or diversity between the groups (*p* > 0.05) (Supplementary Figure 6B and 7A).

Notably, *A. muciniphila* monotherapy increased *Bifidobacterium pseudolongum* abundance but reduced *Alistipes putredinis* and *B. animalis* (*p* < 0.05), effects reversed by Amu + Eha coadministration (Figure 6B–D). *Bifidobacterium pseudolongum* occupied a central influential position in Amu + Eha compared with the other three groups (Figure 6E). Cooccurrence network analysis demonstrated treatment-specific microbial interactions (Supplementary Figure 7C). Despite transient colonization (no stable engraftment post-treatment), both strains induced metabolomic reprogramming, enriching the *α*-linolenic acid, linoleic acid, and glycerophospholipid pathways (Figures 5 and 6, Supplementary Figure 7D–F). Amu + Eha synergistically elevated CD8^+^ tumor-infiltrating lymphocyte (TIL) density by 1.5-fold versus controls (*p* = 0.027; Figure 6H and I, suggesting microbiota-mediated immune potentiation independent of persistent bacterial colonization.

## Discussion

PNI is an independent prognostic indicator for predicting short-term and long-term postoperative outcome in patients undergoing surgery.^18^ Because the cut-off value of PNI differs from context to context,^19^ we determined PNI cut-off value for postoperative lung cancer patients by ROC curve analysis. Our study identified a PNI cutoff of 46.2 as optimal for stratifying NSCLC patients. The high-PNI patients exhibited superior 5-y survival. This cutoff demonstrated higher specificity for stages I and II compared to TNM staging. However, PNI's predictive power diminished in advanced-stage (III) NSCLC, suggesting its utility in the postoperative management for early-stage (I + II) disease. We found that the abundance of SCFA-producing bacteria *E. hallii* and *A. municiphila* were found to be higher in patients with higher PNIs. Oral supplementation with *A. muciniphila* or *E. hallii* or the metabolites butyrate suppressed the growth of cancer cells; moreover, the combination of *A. muciniphila* and *E. Hallii* significantly enhanced the antitumor effect.

Studies have shown that the diversity of the gut microbiome is strongly linked to both nutrition and immune function.^20^ Dysbiosis of the gut microbiome has been considered to be related to nutritional disorders such as obesity, undernutrition and anorexia nervosa.^21^ Healthy gut microbiota metabolizes indigestible macronutrients into short-chain fatty acids and other bioactive compounds.^21^ In our study, the higher abundance of *Akkermansia*, *Eubacterium_hallii_group*, *Ruminococcus*, *Subdoligranulum and Sellimonas* in the high-PNI group suggests that these bacteria are positively correlated with better prognosis through immunologic response and metabolizing dietary nutrients. The gut bacterium *Akkermansia* has an antiobesity effect and may be used as a kind of promising probiotic. It was reported to be the main predictor of total serum levels of free fatty acids.^22^ *Eubacterium halii* is a type of bacteria that is capable of producing SCFAs in the gut. SCFAs are the main metabolites produced by the gut microbiota through the anaerobic fermentation of polysaccharides.^23^ SCFAs produced by the gut microbiota have been shown to play an important role in the maintenance of cellular homeostasis, as they contribute to the regulation of histone deacetylases (HDAC), thereby affecting cell attachment, immune cell migration, cytokine production, chemotaxis, and programmed cell death. Recently, it has been shown that SCFAs can influence the progression of several diseases, such as inflammatory bowel disease (IBD), diabetes, atherosclerosis, and colorectal cancer (CRC).^18^^,^^19^^,^^21^ In lung cancer, one study suggested that sodium butyrate could inhibit lung cancer growth by regulating p21 expression.^22^ Another study suggested that propionate not only inhibits cell growth and induces apoptosis in CRC cell lines, but it also exhibits anti-lung cancer potential by decreasing Survivin expression and increasing p21 expression to activate apoptosis and cell cycle arrest.^22^ This suggests that controlling SCFA levels in the gut by altering the gut microbiota community might be used in the diagnosis and treatment of lung cancer. Studies have found out that acetate is produced by most of the enteric bacteria such as *Lactobacillus* spp., *Bifidobacterium* spp., *Akkermansia muciniphila*, *Bacteroides* spp., *Prevotella* spp., *Ruminococcus* spp., and *Streptococcus* spp. Via the Wood–Ljung–Dahl pathway and acetyl-CoA pathway,^24^ In this study, we identified gut microbiota-associated SCFAs, which may be an indicator for the prognosis in the postoperative patients with NSCLC. The mechanisms of how gut microbiota-associated SCFAs affect the prognosis of postoperative lung cancer patients and possible interventions need to be further investigated.

It is increasingly recognized that gut microbiota composition regulates innate and adaptive immune response through a variety of mechanisms.^25^ The presence or absence of immune cells in the tumor microenvironment has a significant impact on the effectiveness of cancer treatment and patient outcomes. Immune-infiltrated tumors have a high density of immune cells within the tumor, which is often associated with a better prognosis and response to immunotherapy. These tumors are characterized by high levels of TILs, particularly CD8^+^ T cells. The relationship between immune infiltration and the microbiota is a new area of research. Research has shown that the composition of the microbiota in the gut can influence the efficacy of immunotherapy.^26^ A higher abundance of *A. muciniphila* and *Faecalibacterium prausnitzii* has been associated with a more favorable response to immunotherapy in patients with melanoma and lung cancer.^27^^,^^28^ In contrast, a higher abundance of *Bacteroides fragilis* and *Fusobacterium nucleatum* has been associated with a poorer response to immunotherapy.^29^ Immune checkpoint inhibitors (ICIs) of PD-1/PD-L1 can be used for immunotherapy of cancer, and one study reported that resistance to ICIs can be attributed to abnormal gut flora composition, as the use of antibiotics reduced the clinical benefit of using ICIs in patients with advanced cancer. Also transplantation of the fecal microbiota (FMT) from cancer patients who responded to ICIs into sterile or antibiotic-treated mice improved the antitumor effect of PD-1 blockers.^30^ The implantation of *A. muciniphila* in the gut microbiota has emerged as a promising therapeutic strategy due to its potential benefits in metabolic health, immune modulation, and cancer therapy.^31^ However, our findings highlight a critical distinction between two delivery methods: fecal microbiota transplantation (FMT) and oral administration of *A. muciniphila*. Specifically, FMT successfully facilitated the implantation of *A. muciniphila*, while oral administration of the bacterium did not achieve the same effect. Although achieving therapeutic effects in humans and approved for the treatment of recurrent *Clostridium difficile* infection, FMT presents greater challenges and requires further optimization of clinical protocols.^30^ In our study, FMT did not demonstrate tumor shrinkage compared to mice without FMT and caused lower diversity and connection. This discrepancy raises important questions about the donor stool screen. The efficacy and safety of FMT are closely related to donor selection, as the health status of the donor significantly impacts the transplantation outcomes.^32^^,^^33^

The relationship between tumor-infiltrating lymphocytes (TILs) and peripheral blood lymphocytes (PBLs) in cancer patients is complex. Studies have shown that TILs and PBLs share similar T-cell subsets, with CD3+ T cells being predominant in both.^34^ Circulating T cells that share TCRαβ with TILs reflect the effector functions observed in tumors, suggesting a connection between the blood and tumor immune environments.^35^ These findings highlight the potential of using PBLs as a surrogate for monitoring tumor immune responses.

This study has limitations. The prognostic analyses were mainly based on a retrospective cohort, and selection bias and residual confounding cannot be fully excluded even after multivariable adjustment and stage-stratified analyses; therefore, prospective validation is still warranted. The PNI cutoff of 46.2 showed modest specificity in the overall cohort and should be interpreted as a tool for postoperative risk stratification rather than a stand-alone decision threshold. The microbiome discovery cohort was relatively small; we used an independent prospective cohort to provide supportive validation with directionally consistent trends, but larger multicenter studies are still needed. The FMT experiment relied on a single donor per group, which may introduce donor-specific effects; however, recipient-level post-FMT 16S profiling with a No_FMT control, together with qPCR-based engraftment validation and complementary bacterial supplementation experiments, supports biologically relevant microbiota differences after transplantation. Future studies should include multiple donors per group and healthy controls to further strengthen robustness and generalizability.

## Conclusion

In conclusion, our study identifies the PNI as a robust, cost-effective biomarker for stratifying early-stage (I/II) NSCLC patients, with superior specificity over TNM staging in predicting postoperative survival. A potential link between the gut microbiota-SCFA axis and this nutritional-immune interplay has been validated that high-PNI patients exhibited enrichment of *A. muciniphila* and *E. hallii*, whose metabolites (e.g., butyrate) suppressed tumor growth and CD8^+^ T cell activation. Future studies are required to validate these microbial and metabolic signatures in independent, multicenter cohorts and to further elucidate strain-specific mechanisms to optimize personalized therapeutic strategies.

## Supplementary Material

the_second_revised_Supplementary clean.docx the_second_revised_Supplementary clean.docx

Supplementary Figure 6-7.docx Supplementary Figure 6-7.docx

Supplementary Material Revised_Supplemental_Figure1

Supplementary Material Revised_Supplemental_Figure2

Supplementary Material Revised_Supplemental_Figure3

Supplementary Material Revised_Supplemental_Figure4

Supplementary Material Revised_Supplemental_Figure5

## Data Availability

The raw gut microbiome sequencing data from NSCLC patients and murine samples have been deposited in the NCBI Sequence Read Archive under BioProject accessions PRJNA832502 (human) and PRJNA1261867 (murine) (https://dataview.ncbi.nlm.nih.gov/object/PRJNA1261867?reviewer=qrehp3l8ev8keoc5tmbd3dh98o). Metabolomics data are available in the OMIX repository (China National Center for Bioinformation) under accessions OMIX010154 (human) and OMIX010137 (murine).
