# Supplementary material for: Gut microbiota-associated nutritional-immune status predicts prognosis in postoperative NSCLC patients
Source: Gut Microbes. 2026 Apr 3;18(1):2652460. doi: 10.1080/19490976.2026.2652460 (PMC13051591; doi:10.1080/19490976.2026.2652460)
Supplement: the_second_revised_Supplementary clean.docx [file KGMI_A_2652460_SM8615.docx]

**Gut microbiota-associated nutritional-immune status predicts prognosis in postoperative NSCLC patients**

Qian Yu^1,2#^, Anqi Chen^1,2#^, Junqi Yi^1,2#^, Majid iqbal^3^, Ziying Tang^1,2^, Huabo Ge^1,2^, Yan Hu^1,2^, Wenliang Liu^1,2^, Leliang Zheng^3^*, Jingqun Tang^1,2^*, Juanjuan Xiang^1,3^*，

# These authors contribute equally to this study

^1^Hunan Key Laboratory of Early Diagnosis and Precise Treatment of Lung Cancer, The Second Xiangya Hospital, Central South University, Changsha, Hunan, 410013, China

^2^Department of thoracic surgery, the Second Xiangya Hospital, Central South University, Changsha, Hunan, 410013, China

^3^NHC Key Laboratory of Carcinogenesis and the Key Laboratory of Carcinogenesis and Cancer Invasion of the Chinese Ministry of Education, Cancer Research Institute, School of Basic Medical Science, Central South University, Changsha, Hunan, China

***Address correspondence to**：

Juanjuan Xiang [(xiangjj@csu.edu.cn);](mailto:(xiangjj@csu.edu.cn);)

Jingqun [Tang (tangjq@csu.edu.cn)](mailto:Tang(tangjq@csu.edu.cn));

Leliang Zheng(zhengleliang@csu.edu.cn);

Content

[Supplementary Figures 2](#_Toc22265)

[Supplementary Figure 1 | Study design and patient enrollment flowchart. 2](#_Toc31270)

[Supplementary Figure 2: Differences in bacterial abundance at phylum and genus levels. 3](#_Toc12068)

[Supplementary Figure 3: Gut microbiome differences between high-PNI (HPNI) and low-PNI (LPNI) patients in Cohort 2 4](#_Toc12234)

[Supplementary Figure 4: Enterotype stratification in NSCLC patients by PNI status. 5](#_Toc7526)

[Supplementary Figure 5: Recipient gut microbiota structure and differential taxa after FMT. 6](#_Toc23194)

[Supplementary Figure 6: Antitumor effects of butyrates. 7](#_Toc21207)

[Supplementary Figure 7: SCFA-producing bacteria modulate gut microbiota interactions and reshape the fecal metabolome in mice. 8](#_Toc21700)

[Supplementary Tables 9](#_Toc24702)

[Supplementary Table 1: Clinical characteristics of 372 NSCLC patients. 9](#_Toc21632)

[Supplementary Table 2: Clinical characteristics of 25 NSCLC patients(Cohort1). 9](#_Toc4051)

[Supplementary Table 3: Clinical characteristics of 54 NSCLC patients(Cohort2). 11](#_Toc16642)

[Supplementary Table 4: Clinical characteristics of 60 NSCLC patients. 12](#_Toc18546)

[Supplementary Table 5: The primers for 5R 16s rRNA sequencing. 13](#_Toc21467)

[Supplementary Table 6: The primers targeting specific species. 14](#_Toc13414)

[Supplementary Table 7: Independent prognostic factors for postoperative Stage I NSCLC patients. 15](#_Toc4776)

[Supplementary Table 8: The NSCLC patients characteristics of fecal microbiota source. 16](#_Toc8104)

Supplementary Figures

Supplementary Figure 1 | Study design and patient enrollment flowchart.

Supplementary Figure 2: Differences in bacterial abundance at phylum and genus levels.

Supplementary Figure 3: Gut microbiome differences between high-PNI (HPNI) and low-PNI (LPNI) patients in Cohort 2

(A) Venn diagram showing shared and group-specific OTUs between HPNI and LPNI. (B) Alpha-diversity indices (Chao1, observed species, Shannon, and Simpson) compared between groups. (C) PCoA of beta-diversity demonstrating separation of microbial community structure between HPNI and LPNI (PERMANOVA P=0.013). (D–F) Stacked bar plots of taxonomic composition at the phylum (D), genus (E), and species (F) levels. (G–H) LEfSe results shown as a cladogram (G) and LDA score plot (H) highlighting differentially enriched taxa between groups. (I–J) Co-occurrence/correlation networks in the HPNI (I) and LPNI (J) groups (node colors denote phyla; edges represent correlations). (K) Relative abundances of six candidate taxa in HPNI versus LPNI (dot plots overlaid with boxplots; P values are shown): *Eubacterium hallii*, *Akkermansia muciniphila*, *Ruminococcus callidus*, *Haemophilus influenzae*, *Clostridium paraputrificum*, and *Ruminococcus gnavus*.

Supplementary Figure 4: Enterotype stratification in NSCLC patients by PNI status.

(A) PCA analysis of gut microbiota in NSCLC patients (n=25); (B) Calinski-Harabasz index supporting two-cluster optimality. (C) Taxon contributions to Enterotype 1 (*Bacteroidetes*) and Enterotype 2 (*Ruminococcus*). (D) Enterotype distribution across PNI groups. Asterisks denote significance (P < 0.05, *P < 0.01, Student’s t-test).

Supplementary Figure 5: Recipient gut microbiota structure and differential taxa after FMT.

(A) Alpha-diversity indices (Chao1, Observed, Shannon, Simpson) compared among HPNI_FMT, LPNI_FMT, and No_FMT groups. (B) PCoA of beta-diversity showing overall community separation across groups (PERMANOVA P = 0.001). (C) Venn diagram of shared and group-specific OTUs among groups. (D) LEfSe cladogram depicting taxa differentially enriched in each group. (E) LEfSe LDA score plot of discriminative taxa across groups (threshold as indicated). (F) Relative abundance of bacterial phyla in recipient mice. (G) Relative abundance of bacterial genera in recipient mice. (H) Genus-level co-occurrence networks in HPNI_FMT, LPNI_FMT, and No_FMT groups.

Supplementary Figure 6: Antitumor effects of butyrates.

(A) SCFA-producing bacteria experimental workflow; (B) Butyrate experimental workflow. (C) Bioluminescence images of LLC metastases. Quantification of tumor flux (photon/sec/cm²) was shown on the right side. (D)Excised lung (upper) and liver (low) at endpoint; (E) Quantification of tumor flux.; (F) tumor nodule counts; (G) Survival curve.

Supplementary Figure 7: SCFA-producing bacteria modulate gut microbiota interactions and reshape the fecal metabolome in mice.

(A) Shannon diversity index across PBS, Amu, Eha, and Amu+Eha groups. (B) PCoA plots of beta-diversity comparing PBS vs Amu, PBS vs Eha, and PBS vs Amu+Eha (PERMANOVA P values as indicated). (C) Genus-level co-occurrence networks in PBS, Amu, Eha, and Amu+Eha groups. (D–F) Volcano plots of differential fecal metabolites for Amu vs PBS (D), Eha vs PBS (E), and Amu+Eha vs PBS (F). One-way ANOVA with Tukey’s post hoc test was used for (A);

Supplementary Tables

| Supplementary Table 1: Clinical characteristics of 372 NSCLC patients. | | | |
| --- | --- | --- | --- |
| **Variable** | **Case(%)** | **PNI Value** | **P value** |
| **Gender** |  |  | 0.0735 |
| Female | 107(28.76) | 49.16±4.425 |  |
| Male | 265(71.24) | 48.19±4.796 |  |
| **Age(years)** |  |  | 0.3575(F=1.080) |
| ≤50 | 82(22.04) | 49.23±5.090 |  |
| 51-60 | 113(30.38) | 48.42±4.958 |  |
| 61-70 | 138(37.10) | 48.24±4.451 |  |
| ≥71 | 39(10.48) | 47.81±3.915 |  |
| **pTNM classification** |  |  | 0.9555(F=0.1661) |
| ⅠA | 102(27.42) | 48.54±4.570 |  |
| ⅠB | 88(23.65) | 48.29±4.014 |  |
| ⅡA | 26(6.99) | 48.04±5.851 |  |
| ⅡB | 79(21.24) | 48.76±5.083 |  |
| Ⅲ | 77(20.70) | 48.42±4.896 |  |
| **Tumor location** |  |  | 0.1964 |
| Left lung | 173(46.51) | 48.13±4.634 |  |
| Right lung | 199(53.49) | 48.76±4.761 |  |
| **Lobe number** |  |  | 0.4603 |
| Multiple | 14(3.76) | 49.38±5.751 |  |
| Single | 358(96.24) | 48.43±4.667 |  |
| **Histological_type** |  |  | **0.018(F=4.063)** |
| Adenocarcinoma cell carcinoma | 204(54.84) | 49.09±4.660 |  |
| Adenosquamous carcinoma | 15(4.03) | 48.30±5.037 |  |
| Squamous cell carcinoma | 153(41.13) | 47.66±4.645 |  |
| **5-year follow outcome** |  |  | **0.000*** |
| Survival | 214(57.53) | 49.48±4.226 |  |
| Death | 158(42.47) | 47.10±4.985 |  |
| **Total** | 372(100) | 48.47±4.707 |  |
|  |  |  |  |

| Supplementary Table 2: Clinical characteristics of 25 NSCLC patients(Cohort1). | | | |
| --- | --- | --- | --- |
| **Variable** | **Case(%)** | **PNI Value** | **P value** |
| **Gender** |  |  | 0.6333 |
| Female | 17(68.00) | 46.62±2.793 |  |
| Male | 8(32.00) | 45.92±4.489 |  |
| **Age(years)** |  |  | 0.0668(F=2.7730) |
| ≤50 | 7(28.00) | 47.96±4.433 |  |
| 51-60 | 9(36.00) | 47.01±2.446 |  |
| 61-70 | 6(24.00) | 43.38±3.597 |  |
| ≥71 | 3(12.00) | 46.97±2.630 |  |
| **pTNM classification** |  |  | 0.4931 |
| ⅠA | 20(80.00) | 46.16±3.474 |  |
| ＞ⅠA | 5(20.00) | 47.34±2.902 |  |
| **Tumor location** |  |  | 0.0596 |
| Left lung | 9(36.00) | 47.85±2.995 |  |
| Right lung | 16(64.00) | 45.46±3.242 |  |
| **Lobe number** |  |  | 0.5664 |
| Multiple | 3(12.00) | 47.47±2.363 |  |
| Single | 22(88.00) | 46.25±3.474 |  |
| **Histological_type** |  |  | 0.9666 |
| Adenocarcinoma cell carcinoma | 23(92.00) | 46.41±3.358 |  |
| Squamous cell carcinoma | 2(8.00) | 46.30±4.525 |  |
| **Total** | 25(100) | 46.40±3.345 |  |
|  |  |  |  |

| Supplementary Table 3: Clinical characteristics of 54 NSCLC patients(Cohort2). | | | |
| --- | --- | --- | --- |
| **Variable** | **Case(%)** | **PNI Value** | **P value** |
| **Gender** |  |  | 0.6323 |
| Female | 32(59.25) | 49.29±5.084 |  |
| Male | 22(40.75) | 48.63±4.678 |  |
| **Age(years)** |  |  | 0.2593(F=1.381) |
| ≤50 | 15(27.78) | 50.82±3.443 |  |
| 51-60 | 21(38.89) | 48.60±5.736 |  |
| 61-70 | 12(22.22) | 48.85±5.289 |  |
| ≥71 | 6(11.11) | 46.31±2.745 |  |
| **pTNM classification** |  |  | 0.6361 |
| ⅠA | 25(46.30) | 49.36±5.172 |  |
| ＞ⅠA | 29(53.70) | 48.72±4.702 |  |
| **Tumor location** |  |  | 0.0668 |
| Left lung | 24(44.44) | 50.38±4.777 |  |
| Right lung | 30(55.56) | 47.93±4.776 |  |
| **Lobe number** |  |  | 0.177 |
| Multiple | 22(40.75) | 47.93±3.689 |  |
| Single | 32(59.25) | 49.77±5.497 |  |
| **Histological_type** |  |  | 0.6038 |
| Adenocarcinoma cell carcinoma | 44(81.48) | 49.19±3.812 |  |
| Squamous cell carcinoma | 10(18.52) | 48.29±3.898 |  |
| **Total** | 54(100) | 49.02±4.888 |  |
|  |  |  |  |

| Supplementary Table 4: Clinical characteristics of 60 NSCLC patients. | | | |
| --- | --- | --- | --- |
| **Variable** | **Case(%)** | **PNI Value** | **P value** |
| **Gender** |  |  | 0.5728 |
| Female | 32(53.33) | 48.87±4.062 |  |
| Male | 28(46.67) | 48.21±4.965 |  |
| **Age(years)** |  |  | 0.6067(F=0.6173) |
| ≤50 | 13(21.67) | 48.81±4.606 |  |
| 51-60 | 25(41.67) | 49.11±4.497 |  |
| 61-70 | 13(21.67) | 48.52±5.229 |  |
| ≥71 | 9(15.00) | 46.75±3.120 |  |
| **pTNM classification** |  |  | 0.0582 |
| ⅠA | 56(93.33) | 48.27±4.204 |  |
| ＞ⅠA | 4(6.67) | 52.65±6.834 |  |
| **Tumor location** |  |  | 0.7321 |
| Left lung | 18(30.00) | 48.87±5.292 |  |
| Right lung | 42(70.00) | 48.43±4.147 |  |
| **Lobe number** |  |  | 0.3018 |
| Multiple | 5(8.33) | 48.38±4.561 |  |
| Single | 55(91.67) | 50.56±3.111 |  |
| **Histological_type** |  |  | 0.4541 |
| Adenocarcinoma cell carcinoma | 56(90.00) | 48.45±4.237 |  |
| Squamous cell carcinoma | 4(10.00) | 50.20±7.850 |  |
| **Total** | 60(100) | 48.56±4.479 |  |
|  |  |  |  |

| Supplementary Table 5: The primers for 5R 16s rRNA sequencing. | |
| --- | --- |
| **Primer** | **Sequence (5′-3′)** |
| F1 | TGGCGAACGGGTGAGTAA |
| R1 | AGACGTGTGCTCTTCCGATCTCCGTGTCTCAGTCCCARTG |
| F2 | ACTCCTACGGGAGGCAGC |
| R2 | AGACGTGTGCTCTTCCGATCTGTATTACCGCGGCTGCTG |
| F3 | GTGTAGCGGTGRAATGCG |
| R3 | AGACGTGTGCTCTTCCGATCTCCCGTCAATTCMTTTGAGTT |
| F4 | GGAGCATGTGGWTTAATTCGA |
| R4 | AGACGTGTGCTCTTCCGATCTCGTTGCGGGACTTAACCC |
| F5 | GGAGGAAGGTGGGGATGAC |
| R5 | AGACGTGTGCTCTTCCGATCTAAGGCCCGGGAACGTATT |

| Supplementary Table 6: The primers targeting specific species. | | | |
| --- | --- | --- | --- |
| Primer | NR number | Sequence (5′-3′) | Product size (bp) |
| *Akkermansia Muciniphila* | NR_042817.1 | F-GCCGGAATCGCTAGTAATGG | 144 |
|  |  | R-CCTTAGGACCCTGCCTCCTT |  |
| *Clostridium paraputrificum* | NR_026135.1 | F-CCGGTCTCAGTTCGGATTGT | 174 |
|  |  | R-GCTTCCTCCCTTACGGGTT |  |
| *Eubacterium hallii* | NR_118673.1 | F-CGCCGCTAACGCAGTAAGTA | 183 |
|  |  | R-TCTGTCCCGAAGGAAAGCAC |  |
| *Haemophilus influenzae* | NR_044682.2 | F-CCGCGTGAGCGATGAAGTA | 166 |
|  |  | R-GGGCTTTCACATCTGGCTTG |  |
| *Ruminococcus callidus* | NR_029160.1 | F-GAACACAAAGACAGGTGGTGC | 192 |
|  |  | R-CGTGTGTAGCCCAGGTCA |  |
| *Ruminococcus gnavus* | NR_036800.1 | F-ACAGGGGGATAACAGTTGGAAA | 162 |
|  |  | R-ACTGATCGTCGGCTTGGTAG |  |

| Supplementary Table 7: Independent prognostic factors for postoperative Stage I NSCLC patients. | | | | | | |
| --- | --- | --- | --- | --- | --- | --- |
|  | **Univariable Cox regression** | | | **Multivariable Cox regression** | | |
| **Variable** | **HR** | **95% CI** | **P** | **HR** | **95% CI** | **P** |
| **Gender** |  |  |  |  |  |  |
| Male/Female | 2.5550 | 1.2650-5.8800 | **0.0153*** | 1.7710 | 0.7860-4.3690 | 0.1855 |
| **Age** |  |  |  |  |  |  |
| ＜60/≥60 | 0.7007 | 0.3760-1.253 | 0.2431 | 1.1680 | 0.6117-2.1470 | 0.6252 |
| **PNI** |  |  |  |  |  |  |
| ≥46.2/＜46.2 | 0.1940 | 0.1053-0.3452 | **<0.0001*** | 0.1986 | 0.1037-0.3657 | **<0.0001*** |
| **pTNM classification** |  |  |  |  |  |  |
| ⅠA/ⅠB | 0.6142 | 0.3447-1.0780 | 0.0916 | 0.6338 | 0.3509-1.1290 | 0.1237 |
| **Pathological type** |  |  |  |  |  |  |
| Adenocarcinoma/Squamous cell carcinoma and Adenosquamous carcinoma | 0.5573 | 0.3166-0.9788 | **0.0409*** | 0.7670 | 0.4016-1.4390 | 0.4121 |
| **Tumor location** |  |  |  |  |  |  |
| Left lung/Right lung | 1.1147 | 0.6531-2.0170 | 0.6322 | 1.0960 | 0.6124-1.9630 | 0.7557 |
| **Lobe number** |  |  |  |  |  |  |
| Single lobe/Multiple lobe | 0.9552 | 0.2092-16.91 | 0.9638 | 0.6324 | 0.1212-11.6300 | 0.6628 |

| Supplementary Table 8: The NSCLC patients characteristics of fecal microbiota source. | | |
| --- | --- | --- |
|  | **HPNI** | **LPNI** |
| **Gender** | Female | Female |
| **Age** | 33 | 51 |
| **pTNM classification** | T1aN0M0 | T1aN0M0 |
| **Histological_type** | Adenocarcinoma | Adenocarcinoma |
| **Abundance of Eha** | 0.01007146 | 0.00628667 |
| **Abundance of Rgn** | 0.06504859 | 0.20924621 |
| **Abundance of Amu** | 0.00047770 | 0.00001038 |
| **PNI** | 54.8 | 44.2 |
