# Supplementary figures and images for: Gut microbiota-associated nutritional-immune status predicts prognosis in postoperative NSCLC patients
Source: Gut Microbes. 2026 Apr 3;18(1):2652460. doi: 10.1080/19490976.2026.2652460 (PMC13051591; doi:10.1080/19490976.2026.2652460)

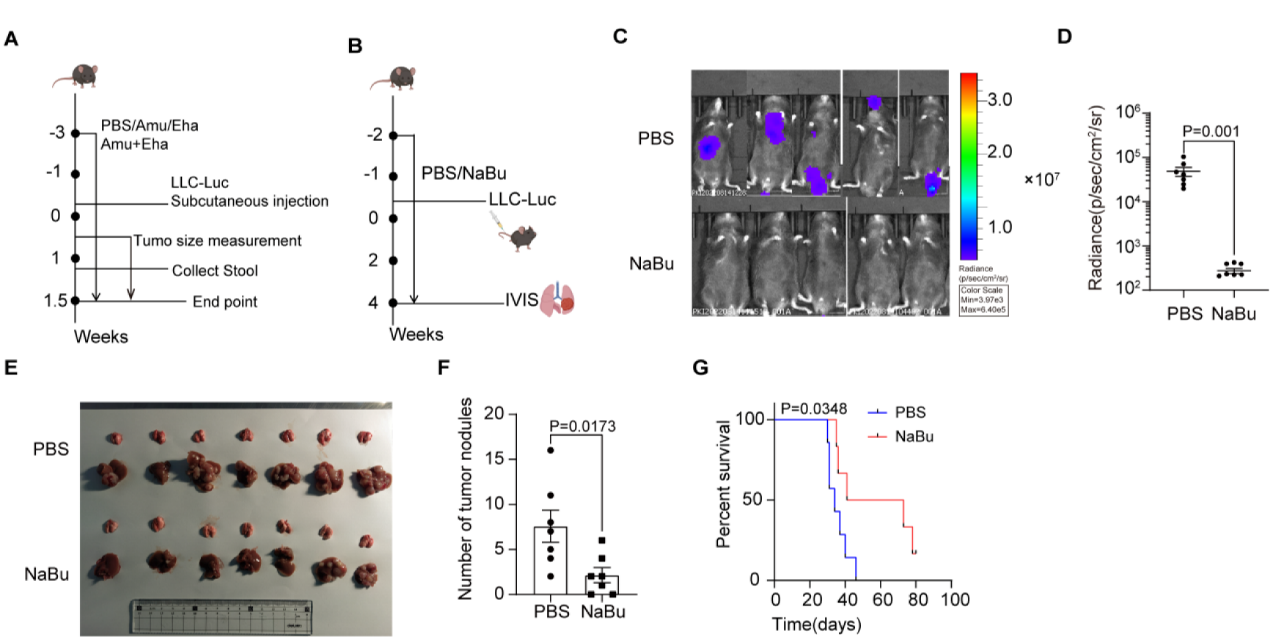


Supplementary Figure 6


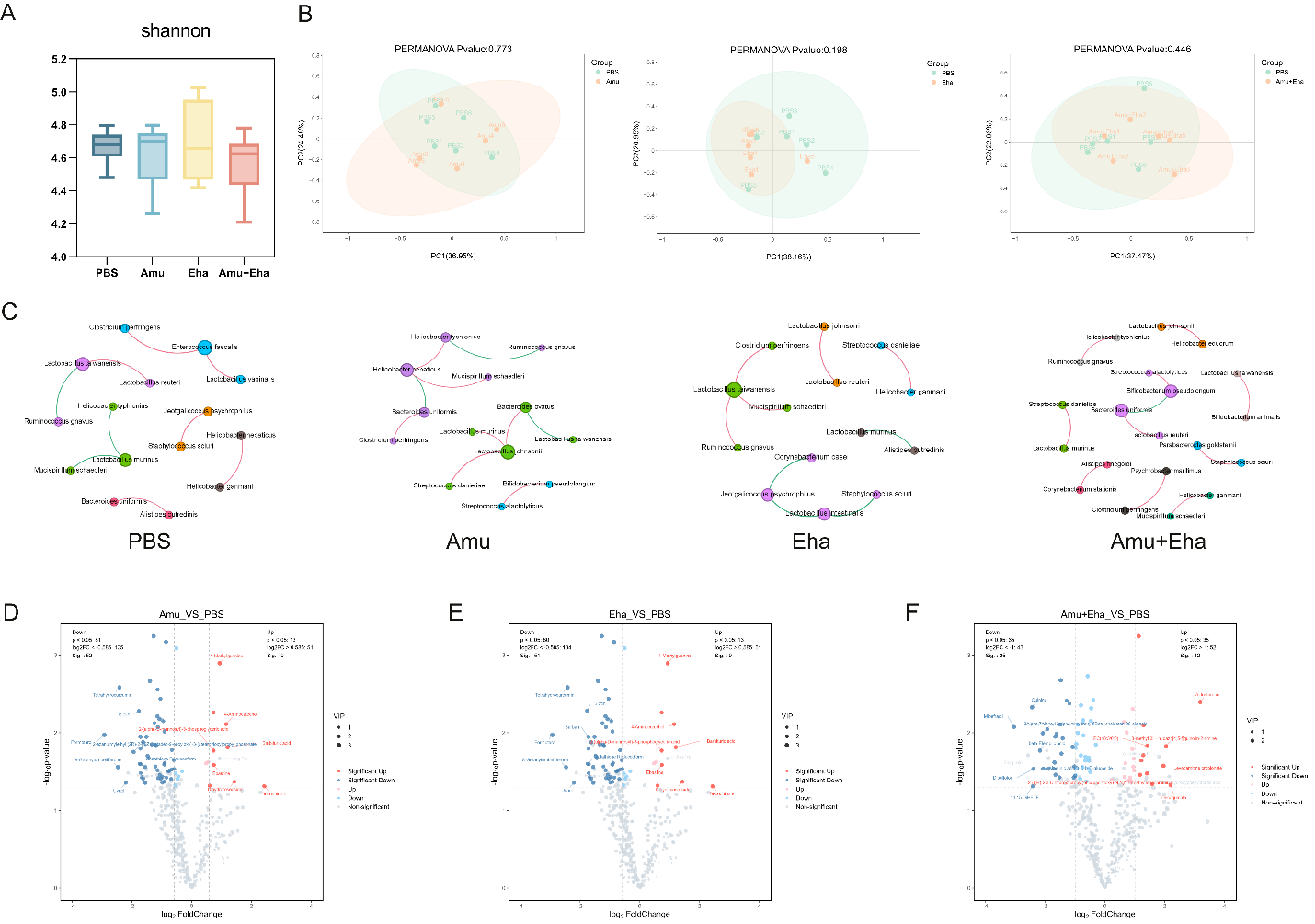


Supplementary Figure 7

Supplement: Supplementary Figure 6-7.docx [file KGMI_A_2652460_SM8614.docx]

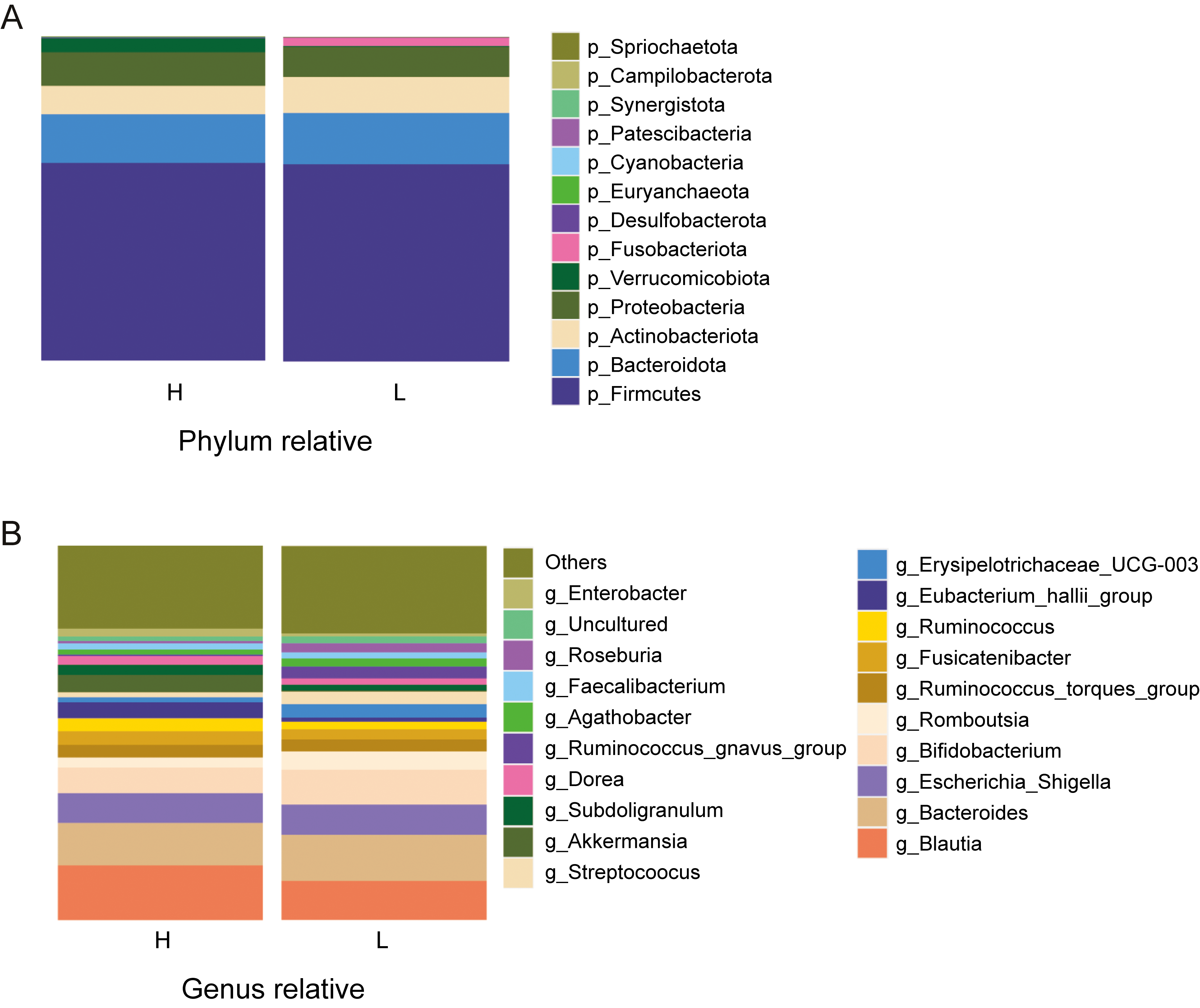

Supplement: Supplementary Material — Revised_Supplemental_Figure2 [file KGMI_A_2652460_SM0011.tif]

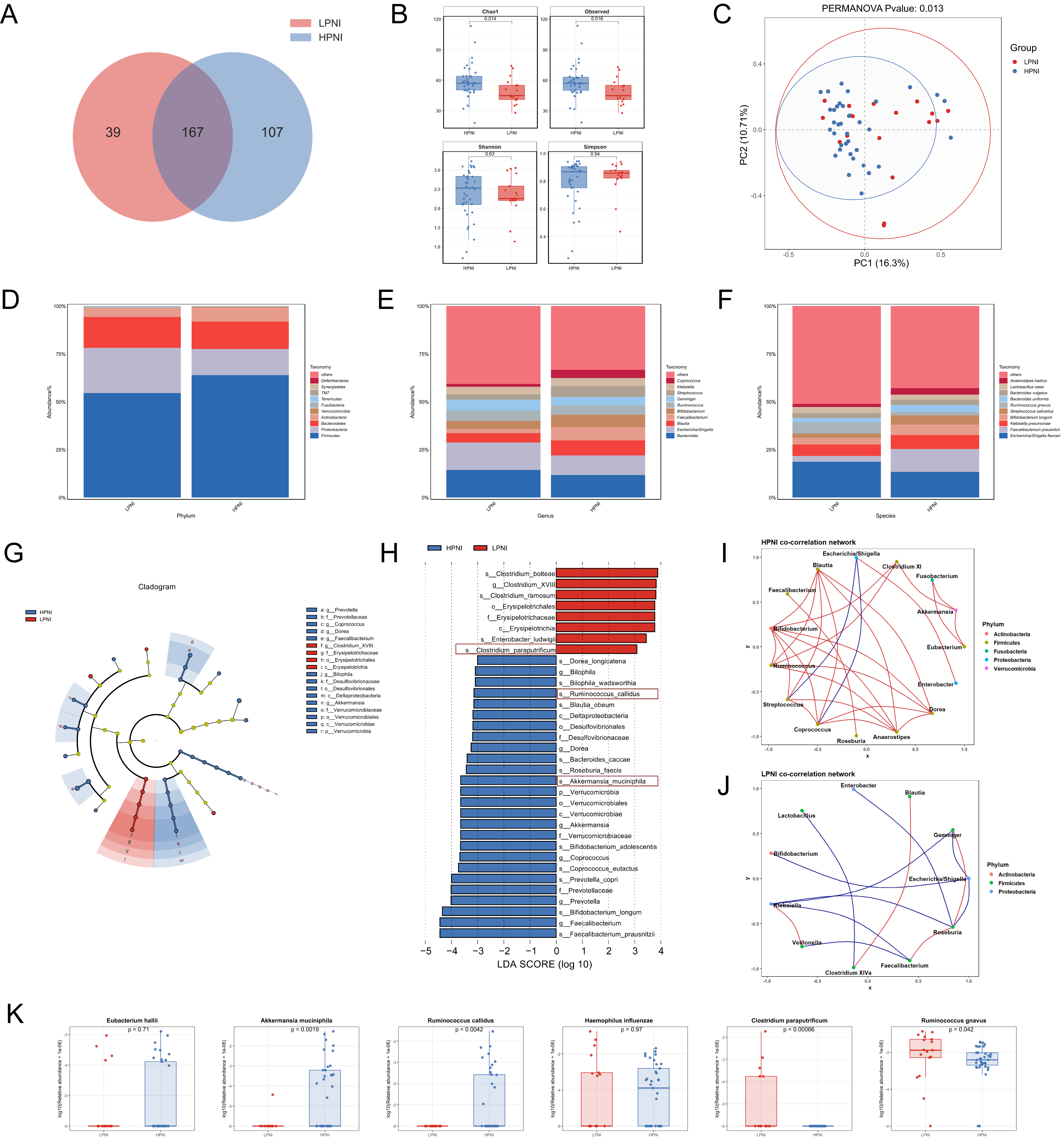

Supplement: Supplementary Material — Revised_Supplemental_Figure3 [file KGMI_A_2652460_SM0010.tif]

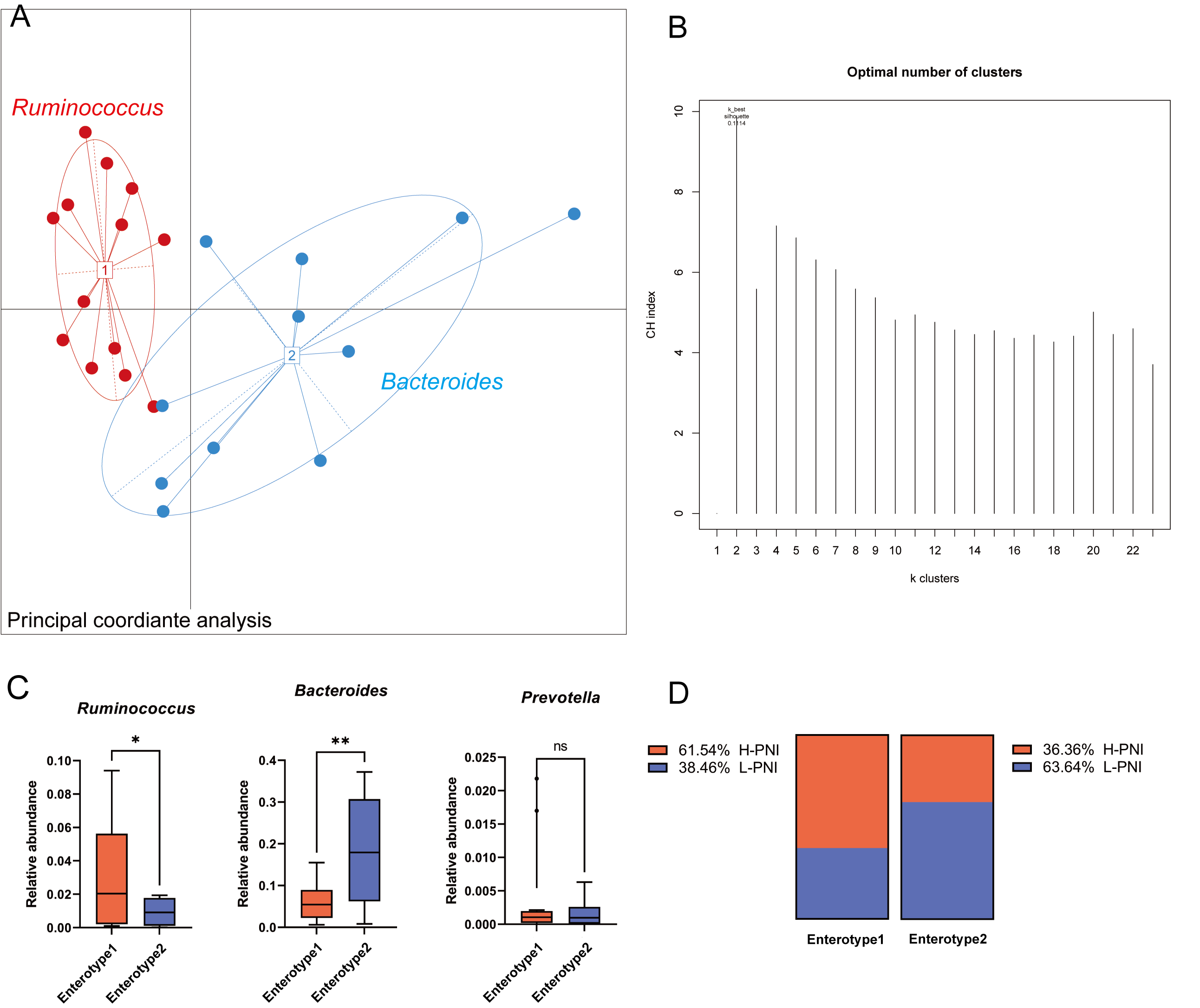

Supplement: Supplementary Material — Revised_Supplemental_Figure4 [file KGMI_A_2652460_SM0007.tif]

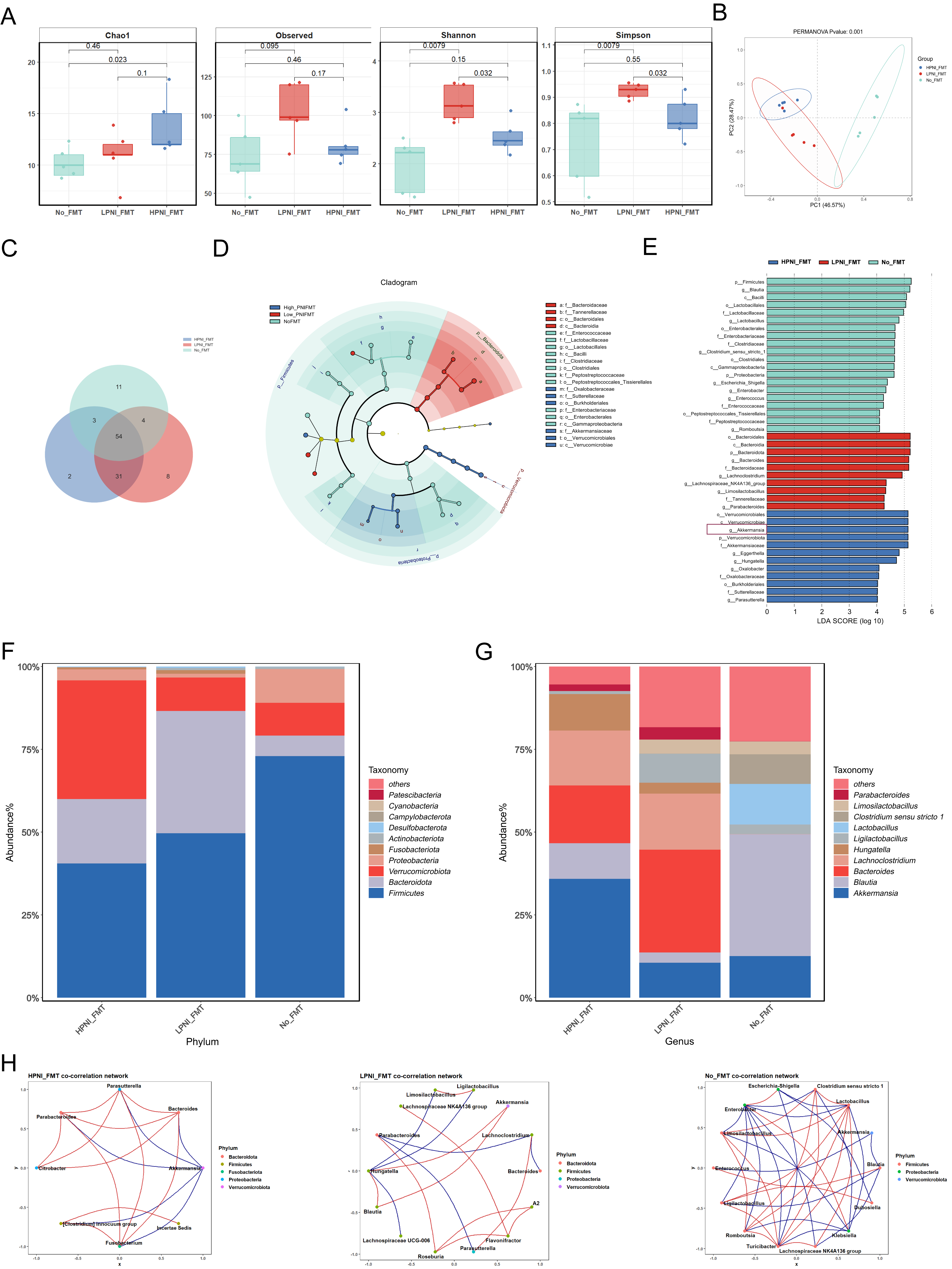

Supplement: Supplementary Material — Revised_Supplemental_Figure5 [file KGMI_A_2652460_SM0004.tif]
